# Comparative 16S rRNA Gene Amplicon Sequencing of the Fecal Microbiome in Pet Dogs and Cats of Different Breeds in Dhaka City, Bangladesh: With Preliminary Insights Into Zoonotic Relevance

**DOI:** 10.1155/ijm/8738439

**Published:** 2026-07-29

**Authors:** Mir Nishat Tasnim Tania, Mirza Synthia Sabrin, Muhammad Abdul Mannan, Md. Mominul Islam, Muhammad Tofazzal Hossain, Md Hafizur Rahman, Md. Rashedul Islam, Sajeda Sultana, Md. Shahdat Hossain, Mahfuzul Islam

**Affiliations:** ^1^ Department of Microbiology and Parasitology, Sher-e-Bangla Agricultural University, Dhaka, Bangladesh, sau.edu.bd; ^2^ Department of Pathology, Sher-e-Bangla Agricultural University, Dhaka, Bangladesh, sau.edu.bd; ^3^ Department of Microbiology and Hygiene, Bangladesh Agricultural University, Mymensingh, Bangladesh, bau.edu.bd; ^4^ AMR Reference Laboratory (Research), Animal Health Research Division, Bangladesh Livestock Research Institute, Dhaka, Bangladesh, blri.gov.bd; ^5^ Department of Surgery and Theriogenology, Sher-e-Bangla Agricultural University, Dhaka, Bangladesh, sau.edu.bd; ^6^ Fisheries Biotechnology Division, National Institutes of Biotechnology, Dhaka, Bangladesh

**Keywords:** 16s rRNA metagenomics, feces, microbial diversity, pets, zoonoses

## Abstract

Dogs and cats are the most commonly kept pets, and the popularity of different breeds of them continues to increase in Dhaka City, Bangladesh. Pets naturally harbor a diverse gut microbiome that plays a significant role in digestion, immunity, and overall health. Although pets provide valuable companionship, their feces may occasionally harbor bacteria with zoonotic potential. However, little is known about the fecal microbial diversity and its zoonotic relevance in Bangladesh. This study investigated the diversity of pets′ fecal microbiome using 16S rRNA metagenomics and explored the zoonotic bacterial taxa. Fecal samples were collected from 24 apparently healthy pets, including 12 dogs and 12 cats, from randomly selected households in Dhaka City. High‐throughput sequencing revealed a diverse microbial community comprising 1,148 amplicon sequence variants (ASVs) distributed across 20 phyla and 258 genera. Although cats showed slightly higher microbial richness and diversity, both species shared common bacterial phyla such as Firmicutes, Proteobacteria, Actinobacteria, and Bacteroidetes. Relative abundance of bacterial taxa varied between pet species and among breeds rather than the presence of distinct microbial groups. Furthermore, *Enterococcus cecorum*, *Schaalia canis*, *Campylobacter helveticus*, and *Sutterella wadsworthensis* were the explored bacterial taxa with zoonotic relevance rather than a direct assessment of zoonotic risk; however, they were very low in number than the dominating bacteria. This study provides the first 16S rRNA‐based metagenomic snapshot of the fecal microbiome of 24 urban pets in Bangladesh and highlights the need for routine microbial surveillance and public awareness about zoonoses.

## 1. Introduction

The role of dogs and cats in human society has shifted dramatically over the past few decades. In the past they were mainly kept for guarding, controlling rodents, or serving as barn companions, but now they are regarded as members of the family [1]. Many pets share close physical contact with their owners like living indoors, sleeping on beds, or even licking faces [2]. This growing human–animal bond, often described as anthropomorphism or the humanization of pets, reflects a recognition of their emotional and sentient qualities. Beyond companionship, pets can provide measurable health benefits, such as lowering stress and improving psychological well‐being [3, 4].

Mammalian gut harbors a diverse group of microbes that serves essential functions in host nutrition, metabolism, immune development, and disease resistance [5, 6]. In companion animals, the gut microbes are shaped by several factors, including host species, breed, age, genetics, diet, environment, and health status [7, 8]. Gut microbes are continuously shed into the environment through feces, creating opportunities for animal and human transmission of microbes [9]. Several breeds of dogs and cats are commonly reared as pets in the urban areas of Bangladesh; however, little is known regarding the species and breed‐related gut and/or fecal microbial diversity.

Despite benefits, close contact with animals comes with public health risks. Cats and dogs engage in behaviors such as licking their genital area, eating feces (coprophagia), or rolling in animal remains. These activities expose them to a wide range of microorganisms, some of which have zoonotic potential [10]. These pathogens can be transmitted to humans through direct interactions like bites, scratches, or licking, or indirectly through environmental contamination [11]. Bacterial zoonoses are especially common and include conditions such as pasteurellosis, cat scratch disease, and gastrointestinal infections caused by *Salmonella* or *Campylobacter*. Other concerning pathogens include *Leptospira*, methicillin‐resistant *Staphylococcus aureus* (MRSA), and agents of tuberculosis [11]. For vulnerable populations such as children, the elderly, or immunocompromised individuals, these infections can result in severe disease. In addition to infectious risks, pet ownership may also lead to allergic reactions, injuries from bites and scratches, or exposure to vector‐borne diseases [12].

These concerns are not limited to wealthier nations. In many developing countries, dogs and cats play equally important roles as companions or community animals, yet often receive little or no veterinary care. This lack of medical oversight increases the likelihood of disease transmission [13]. Free‐roaming or community owned animals, in particular, may act as large, uncontrolled reservoirs of infectious agents. Their interactions with wildlife further increase the risk of spreading pathogens, sometimes indirectly through arthropod vectors. Even in urban environments, domestic pets may come into contact with wild species such as rodents or urban foxes, creating opportunities for microbial exchange [10, 14]. In Bangladesh, and especially in Dhaka City, scientific attention to the zoonotic risks posed by companion animals has been limited. A handful of studies have reported the presence of *Staphylococcus aureus*, *Escherichia coli*, and *Salmonella* spp. in cats, including pathogenic strains like enteropathogenic *E. coli* [15, 16]. Since both dogs and cats shed microorganisms in their feces, these pathogens can easily contaminate the household environment and potentially infect humans. Existing poor knowledge and good attitude of dog and cat owners in Bangladesh regarding zoonotic diseases have been reported [17], further strengthening the need for screening fecal microbes with zoonotic potential.

Thus, the present study investigates the 16S rRNA‐based metagenomic analysis of the fecal microbiomes of pet dogs and cats in Dhaka City, Bangladesh, with preliminary insights into zoonotic relevance.

## 2. Material and Methods

### 2.1. Ethical Approval

Ethical approval is not mandatory for this type of research. Prior consent was taken from the owners of the pets, and during sampling, pets were handled carefully.

### 2.2. Sample Collection

This study was designed as a cross‐sectional comparative observational study with stratified sampling of pet dogs and cats of different breeds in Dhaka City, Bangladesh. A total of 24 fecal samples were aseptically collected in sterile conical tubes from randomly selected apparently healthy pet animals (12 from dogs and 12 from cats) immediately after defecation during the period from July 2024 to June 2025. Pets were selected from different households based on the owners′ willingness to participate. Samples were collected from local breed (DLB) (*n* = 4), golden retriever (DRG) (*n* = 4), and German shepherd (DSG) (*n* = 4) of dogs, whereas domestic shorthair (CDS) (*n* = 4), Persian (CP) (*n* = 4), and mixed breed (CMB) (*n* = 4) of cats. Immediately after collection, samples were transferred to the respective laboratory and were stored at −20°C for further analysis.

### 2.3. Extraction of DNA and Amplicon Generation

The phenol chloroform method was used to extract all of the genomic DNA from the samples. A 1% agarose gel was used to measure the concentration and purity of DNA. The 16S rRNA gene of distinct regions 16S V3–V4 was amplified using a particular primer (341F: CCTAYGGGRBGCASCAG and 806R: GGACTACNNGGGTATCTAAT) [18]. Every PCR reaction was conducted using approximately 10 ng of template DNA, 0.2 *μ*M of forward and reverse primers, and 15 *μ*L of Phusion High‐Fidelity PCR Master Mix (New England Biolabs). Initially, the material was denatured at 98°C for 1 min. This was followed by 30 cycles of denaturation at 98°C for 10 s, annealing at 50°C for 30 s, and elongation at 72°C for 30 s. At last, final extension was performed at 72°C for 5 min. The PCR products were mixed with the same volume of 1x loading buffer containing SYB green and electrophoresis using 2% agarose gel for detection. The PCR products were combined in ratios of equal density. The Qiagen Gel Extraction Kit (Qiagen, Germany) was then used to purify the set of PCR products.

### 2.4. Library Preparation, Sequencing, and Quality Control

Amplicon metagenomic analysis was carried out at Beijing, China′s Novogene Bioinformatics Technology Co., Ltd. Index codes were added after the TruSeq DNA PCR‐Free Sample Preparation Kit (Illumina, United States) was used to create sequencing libraries in accordance with the manufacturer′s instructions. The Agilent Bioanalyzer 2100 system and the Qubit 2.0 Fluorometer (Thermo Scientific) were used to evaluate the library′s quality. After the library was finally sequenced using an Illumina NovaSeq 6000 platform (PE250), (250X2) bp paired‐end reads were produced for the 16S rRNA gene′s V3–V4 variable regions. After being truncated by removing the primer sequence and barcode, paired‐end reads were demultiplexed and allocated to samples according to their distinct barcode. A high‐speed and accurate analysis tool called FLASH (VI.2.7,http://ccb.jhu.edu/software/FLASH/) [19] was used to merge paired‐end reads when at least some of the bases overlapped the read generated from the opposite end of the same DNA fragment. The raw sequencing reads were quality‐filtered with the QIIME2 (V1.9.1) and screened for chimeras [20–22].

### 2.5. Data Filtering and Analysis Process

The raw data were first spliced and filtered to produce more accurate and trustworthy clean data, which was then used to improve the accuracy and dependability of the information analysis results. To obtain the final ASVs, denoise using DADA2 and remove sequences with an abundance of less than two across the entire dataset (i.e., all samples combined) to filter out low‐confidence ASVs that may be due to sequencing errors or background noise. On the one hand, species annotation was done on each ASV′s representative sequence, yielding the species‐based abundance distribution and related species information [23]. According to the annotation results of ASVs and the characteristic tables of each sample, species abundance tables at the kingdom, phylum, class, order, family, genus, and species levels were obtained. These abundance tables with annotation information were the core content of amplicon analysis. According to different experimental purposes, one or several key species were screened from the species abundance table at each taxonomic level (usually focusing on the phylum and genus level), combined with different samples (groups) species composition and difference analysis, cluster analysis and other results for in‐depth research.

In order to determine species richness and evenness information within the sample, as well as common and unique ASVs information among various samples or groups (species and breeds), ASVs were simultaneously analyzed using abundance, alpha‐diversity calculation (Chao1 richness, Shannon diversity index, and Simpson diversity index) (QIIME2 [V1.9.1]), Venn diagram (R [V4.0.3]), and flower diagram (perl [V5.26.2]). Furthermore, alpha diversity was compared between pet species using Wilcoxon rank‐sum test. Beta diversity was assessed using Bray–Curtis dissimilarities, visualized by Principal Coordinates Analysis (PCoA), and group differences were tested using PERMANOVA ([QIIME2 (V1.9.1) and R (V4.0.3)]). Heatmaps were produced for the abundant taxa (R [V4.0.3]).

### 2.6. Assessment of Zoonotic Relevance

The presence of bacterial taxa previously associated with zoonotic infections was assessed as a measure of zoonotic relevance rather than as evidence of confirmed zoonotic risk.

## 3. Results

### 3.1. Sequence Reads and ASV Classification of the Fecal Microbiome of Pets

**Figure 1:**
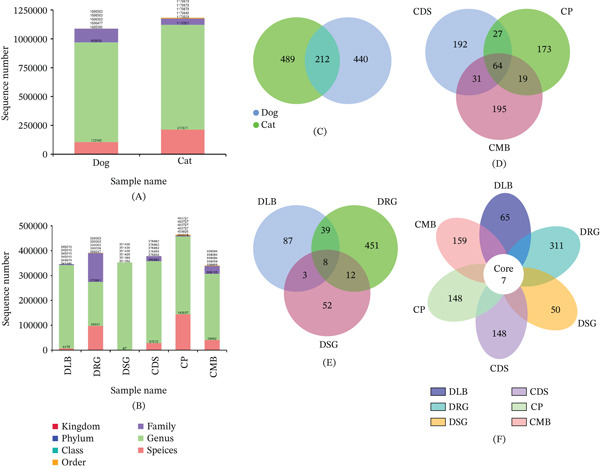
Sequence reads and ASV classification of the faecal microbiome of dogs and cats. (A) Sequence number of dogs and cats. (B) Sequence number of different breeds of pets. (C) Venn diagram of ASVs for dogs and cats. (D) Venn diagram of ASVs for cat breeds. (E) Venn diagram of ASVs for dog breeds. (F) Flower diagram of ASVs for all groups of pets. Each circle in the figure represents a species or breed; the numbers in circles and circle overlap represent the number of shared or unique ASVs in different groups. CDS, cat (domestic shorthair); CMB, cat (mixed breed); CP, cat (Persian); DLB, dog (local breed); DRG, dog (golden retriever); DSG, dog (German shepherd).

We analyzed fecal samples of 12 dogs and 12 cats using an Illumina Novaseq sequencing platform. A total of 2,266,176 high‐quality sequence reads (1,086,503 for dogs and 1,179,673 for cats, whereas 345,010 for DLB, 390,063 for DRG, 351,430 for DSG, 376,862 for CDS, 463,727 for CP, and 339,084 for CMB) were obtained from the 24 apparently healthy pets (Figure 1A,B). A total of 1,148 bacterial ASVs classified into 20 phyla, 34 classes, 85 orders, 137 families, 258 genera, and 138 species were obtained. All the unidentified taxa were designated as “others.” Among the 1,148 bacterial ASVs, a total of 212 ASVs were common in both pets; 489 and 440 ASVs were unique for dogs and cats, respectively (Figure 1C). Similarly, varied number of unique ASVs were observed in different cats and dogs breeds (Figure 1D,E). According to the flower diagram, only 7 ASVs were shared among the pet breeds in this study, whereas 311, 159, 148, 148, 65, and 50 unique ASVs were observed in DRG, CMB, CDS, CP, DLB, and DSG, respectively (Figure 1F).

### 3.2. Richness and Diversity of the Fecal Microbiome of Pets

**Figure 2:**
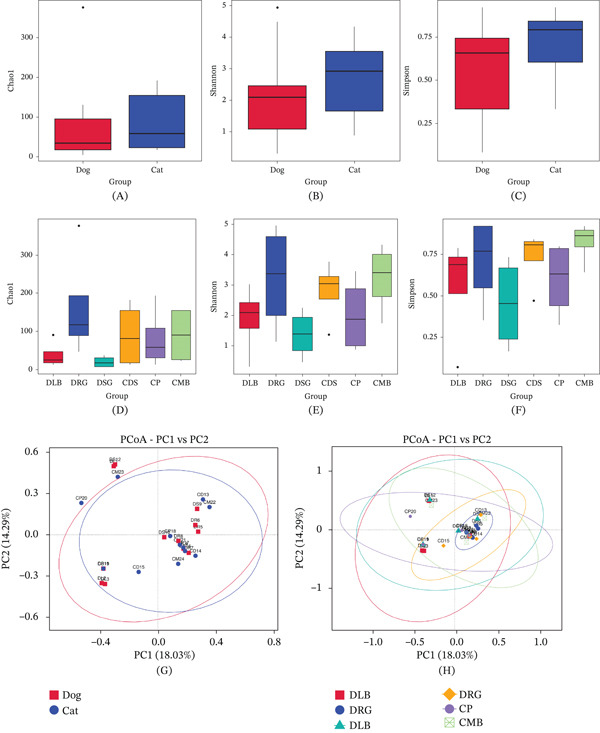
Alpha and beta diversity and microbial community structures across pets. (A) The observed richness (Chao1), (B) Shannon diversity index, and (C) Simpson diversity index of microbial communities in samples from dogs and cats. (D) The observed richness (Chao1), (E) Shannon diversity index, and (F) Simpson diversity index of microbial communities in samples from different breeds of pets. Principal coordinates analysis (PCoA) was performed based on Bray–Curtis dissimilarity for (G) dogs and cats and (H) different breeds of pets. CDS, cat (domestic shorthair); CMB, cat (mixed breed); CP, cat (Persian); DLB, dog (local breed); DRG, dog (golden retriever); DSG, dog (German shepherd).

Alpha and beta diversity and microbial community structures across pets are presented in Figure 2. The Chao1 (observed richness), Shannon, and Simpson diversity were higher in cats compared with dogs (Figure 2A–C). Among pet breeds, DRG had the highest observed richness, Shannon and Simpson diversity followed by CMB and CDS (Figure 2D–F). PCoA, which was performed based on Bray–Curtis dissimilarity, revealed no clustering between dogs and cats and different breeds of pets (Figure 2G,H).

### 3.3. Microbial Community Composition in the Feces of Pets

**Figure 3:**
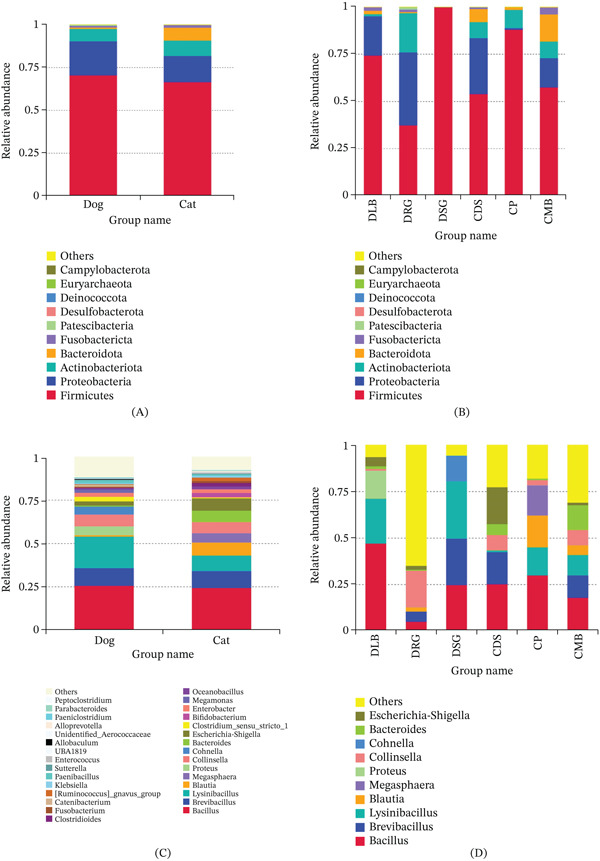
Relative abundance at the phylum and genus level of dogs and cats and different breeds of pets. (A) Phylum level of pets, (B) phylum level of different breeds of pets, (C) genus level of pets, and (D) genus level of different breeds of pets. CDS, cat (domestic shorthair); CMB, cat (mixed breed); CP, cat (Persian); DLB, dog (local breed); DRG, dog (golden retriever); DSG, dog (German shepherd).

**Figure 4:**
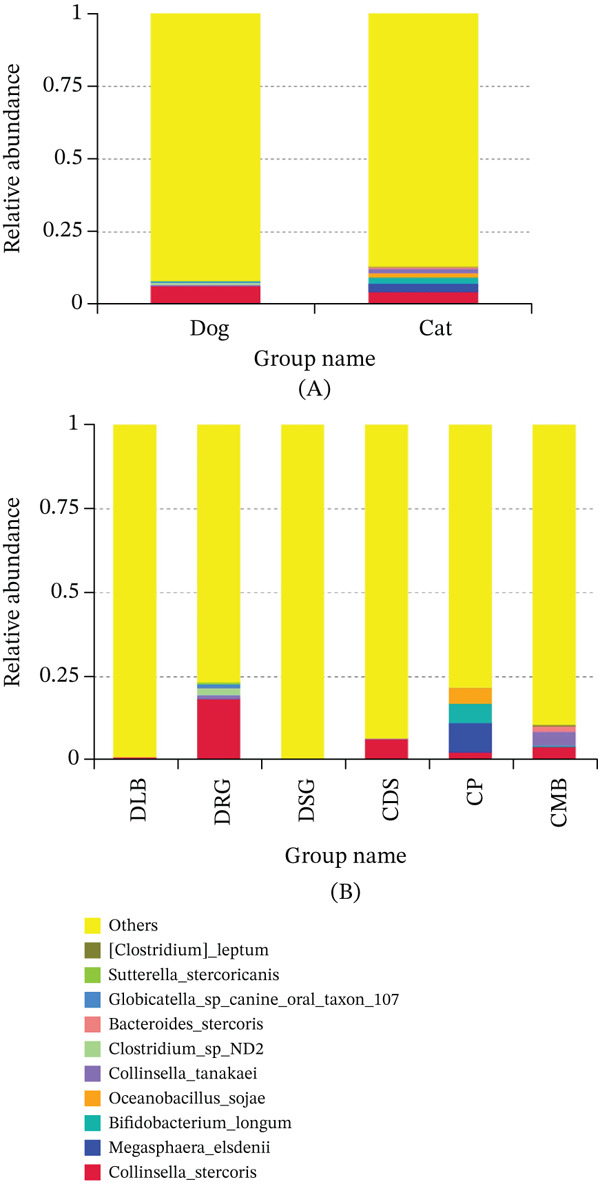
Relative abundance at the species level of (A) dogs and cats and (B) different breeds of pets. CDS, cat (domestic shorthair); CMB, cat (mixed breed); CP, cat (Persian); DLB, dog (local breed); DRG, dog (golden retriever); DSG, dog (German shepherd).

The following five phyla were predominant (≥ 1% of total sequences), accounting for over 99.00% of total 16S rRNA sequences in the pets (dogs and cats): Firmicutes (70.63% vs. 66.62%), Proteobacteria (19.78% vs. 15.19%), Actinobacteria (7.34% vs. 9.08%), Bacteroidetes (0.79% vs. 7.49%), and Fusobacteria (1.03% vs. 1.52%) (Figure 3A). The highest abundance of Firmicutes was recorded in DSG; however, Proteobacteria was most prevalent in DRG (Figure 3B). At the genus level, 12 and 15 taxa were predominant (≥ 1% of the total sequences) in dogs and cats, respectively. Among them, *Bacillus*, *Lysinibacillus*, and *Brevibacillus* were more abundant genera in both dogs and cats; however, they were higher in dogs (Figure 3C). In terms of breed, *Bacillus* was more abundant in DLB, whereas *Lysinibacillus* and *Brevibacillus* were in DSG (Figure 3D). Although a large proportion of undefined bacterial species was observed, which categories as “others” (91.41% for dogs and 86.02% for cats), *Collinsella stercoris* was the most prevalent bacterial species in both species (6.48% vs. 4.45% for dogs and cats), and the highest abundance was observed in DRG (Figure 4A,B).

### 3.4. Specific Fecal Biomarkers of Pets′ Breeds

**Figure 5:**
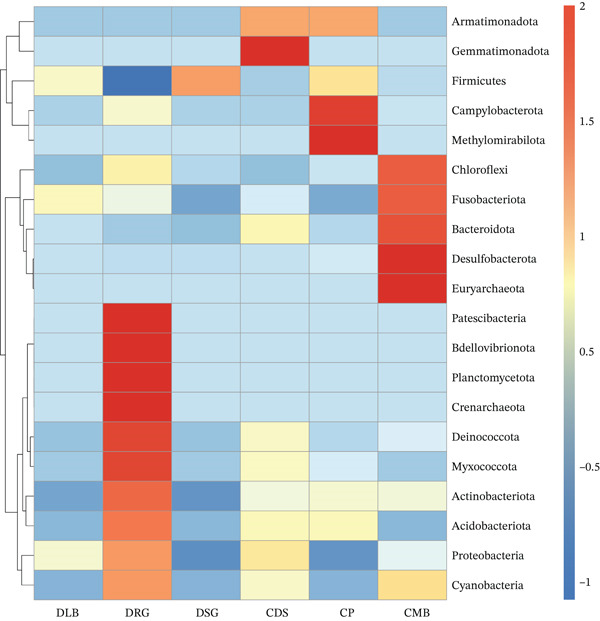
Heatmap of 20 most abundant fecal microbial phyla of different breeds of pets. Color intensity represents *z*‐score–standardized relative abundance, with red indicating relatively higher abundance and blue indicating relatively lower abundance compared with the overall mean abundance of each taxon. CDS, cat (domestic shorthair); CMB, cat (mixed breed); CP, cat (Persian); DLB, dog (local breed); DRG, dog (golden retriever); DSG, dog (German shepherd).

**Figure 6:**
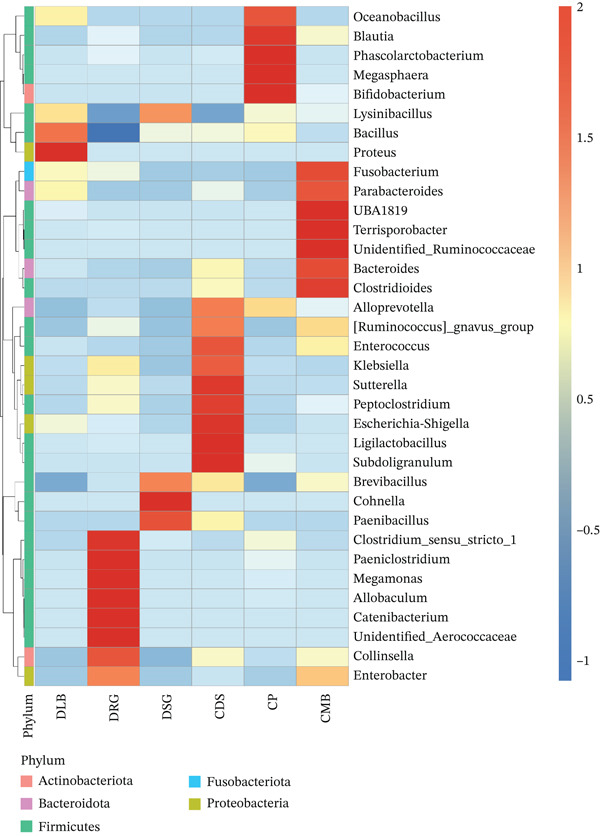
Heatmap of 35 most abundant fecal microbial genera of different breeds of pets. Color intensity represents *z*‐score–standardized relative abundance, with red indicating relatively higher abundance and blue indicating relatively lower abundance compared with the overall mean abundance of each taxon. CDS, cat (domestic shorthair); CMB, cat (mixed breed); CP, cat (Persian); DLB, dog (local breed); DRG, dog (golden retriever); DSG, dog (German shepherd).

**Figure 7:**
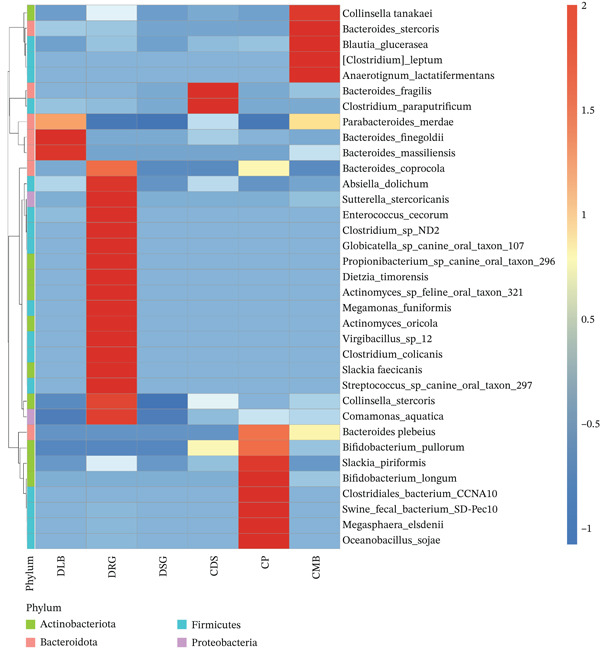
Heatmap of 35 most abundant fecal microbial species of different breeds of pets. Color intensity represents *z*‐score–standardized relative abundance, with red indicating relatively higher abundance and blue indicating relatively lower abundance compared with the overall mean abundance of each taxon. CDS, cat (domestic shorthair); CMB, cat (mixed breed); CP, cat (Persian); DLB, dog (local breed); DRG, dog (golden retriever); DSG, dog (German shepherd).

Heatmap of most abundant fecal microbial 20 phyla, 35 genera, and 35 species are presented in Figures 5, 6, and 7. The phyla Crenarchaeota, Planctomycetota, Bdellovibrionota, and Patescibacteria were more abundant in DRG compared with other pet breeds. In contrast, Euryarchaeota and Desulfobacterota were more abundant in CMB, Methylomirabilota and Campylobacterota in CP, and Gemmatimonadota in CDS. In terms of genera, DRG had higher abundance of genera unidentified_Aerococcaceae, *Catenibacterium, Allobaculum, Megamonas, Paeniclostridium,* and *Clostridium_sensu_stricto_1. Cohnella* was more abundant in DSG. The genera *Subdoligranulum, Ligilactobacillus, Escherichia-Shigella, Peptochlostridium, and Sutterella* were more prevalent in CDS. However*, Clostridioides,* unidentified_Ruminococcaceae*, Terrisporobacter*, and UBA1819 were more abundant in CMB. DLB had higher abundant bacterial genera of *Proteus* and *Bacillus*. In contrast, the genera *Bifidobacterium*, *Megasphaera*, *Phascolarctobacteriu*, and *Blautia* were highly prevalent in CP.

In species level, CP had the highest abundance of bacterial species of *C*. *stercoris*, *Megasphaera elsdenii*, swine_fecal_bacterium_SD‐Pec10, *Clostridiales bacterium*_CCNA10, *Bifidobacterium longum*, and *Slackia piriformis*. The following species were more prevalent in DRG: *Streptococcus* sp_canine_oral_taxon_297, *Slackia faecicanis*, *Clostridium colicanis*, *Virgibacillus* sp_12, *Actinomyces oricola*, *Megamonas funiformis*, *Actinomyces* sp_feline_oral_taxon_321, *Dietzia timorensis*, *Propionibacterium* sp_canine_oral_taxon_296, *Globicatella* sp_canine_oral_taxon_107, *Clostridium* sp_ND2, *Enterococcus cecorum*, *Sutterella stercoricanis*, and *Absiella dolichum*. In contrast, *Bacteroides massiliensis* and *Bacteroides finegoldii* were more abundant in DLB, and *Clostridium paraputrificum* and *Bacteroides fragilis* in CDS. However, CMB had the highest abundance of the following bacterial species: *Anaerotignum lactatifermentans*, (*Clostridium*) *leptum*, *Blautia glucerasea*, *Bacteroides stercoris*, and *Collinsella tanakaei*.

### 3.5. Zoonotic Relevance of Pets′ Feces

Bacterial taxa previously associated with zoonotic infections were assessed as indicators of zoonotic relevance rather than of confirmed zoonotic risk. Among the zoonotic bacterial taxa, the read counts of *E*. *cecorum, Schaalia canis*, and *Corynebacterium auriscanis* were higher in DRG (Table 1). In contrast, a higher read count of *Campylobacter helveticus* was observed in CP, and the same of *Streptococcus dysgalactiae* in CMB (Table 1). Although, DSG had no detected zoonotic bacterial taxa in their feces, the reads of *B*. *massiliensis* was the abundant bacterial taxa in DLB (Table 1).

**Table 1:** List of bacterial taxa of pets with zoonotic relevance.

Bacterial taxa	Number of ASV reads detected						Zoonotic relevance
	DLB	DRG	DSG	CDS	CP	CMB	
*Enterococcus cecorum*	4	133	0	0	0	0	Yes
*Schaalia canis*	7	58	0	0	4	0	Yes
*Campylobacter helveticus*	0	0	0	0	41	0	Yes
*Sutterella wadsworthensis*	0	0	0	38	0	0	Yes
*Corynebacterium auriscanis*	0	21	0	0	0	0	Yes
*Streptococcus dysgalactiae*	0	0	0	0	0	9	Yes
*Streptococcus canis*	0	4	0	0	0	0	Yes
*Enterococcus casseliflavus*	0	2	0	0	0	0	Yes
*Lawsonella clevelandensis*	0	2	0	0	0	0	Yes
*Campylobacter gracilis*	0	2	0	0	0	0	Yes
*Bacteroides massiliensis*	132	0	0	0	0	21	Not established yet
*Corynebacterium urealyticum*	5	0	0	0	0	0	Not established yet

Abbreviations: CDS, cat (domestic shorthair); CMB, cat (mixed breed); CP, cat (Persian); DLB, dog (local breed); DRG, dog (golden retriever); DSG, dog (German shepherd).

## 4. Discussion

This study compared the relative abundance of fecal microbiota in apparently healthy dogs and cats using 16S rRNA‐based metagenomic profiling, providing novel insights into species‐ and breed‐level microbial variation. Fecal samples collected from 12 dogs and 12 cats in Dhaka, Bangladesh, were analyzed. Overall, cats exhibited greater alpha diversity (Chao1/observed richness, Shannon, and Simpson indices) compared with dogs, indicating a tendency toward a more diverse fecal microbial diversity. Previous research comparing the gut microbiota of companion animals has shown similar patterns, which may be due to variations in host physiology, gastrointestinal ecology, feeding patterns, and evolutionary history of dogs and cats [24, 25]. Among dogs, DRG showed the highest diversity values, suggesting probable breed‐associated differences in gut microbial composition. However, alpha‐diversity differences between species were not statistically significant, indicating that the fecal microbiota of dogs and cats exhibited broadly comparable diversity patterns in the present study. This nonsignificant finding may be attributed to the limited sample size and the substantial interindividual variability in the gut microbiota [25]. Beta diversity analysis corroborated the alpha‐diversity findings, with Bray–Curtis PCoA showing substantial overlap among samples and no species‐ or breed‐specific clustering, which is consistent with the findings of Langon et al. [24]. This indicates that individual variation may have a stronger impact on gut microbial shaping than the host species or breed.

At the phylum level, Firmicutes, Bacteroidetes, Fusobacteria, Proteobacteria, and Actinobacteria dominated in both cats and dogs, consistent with previous studies [26–31]. These phyla also form the core feline and canine fecal microbiome and are associated with the metabolic functions, including carbohydrate fermentation, short‐chain fatty acid production, protein metabolism, and maintenance of intestinal homeostasis [24, 32]. Relative abundance analysis showed higher Actinobacteria and Bacteroidetes in cats, and higher Firmicutes and Proteobacteria in dogs. Similar patterns have been reported earlier and may reflect differences in dietary habits, digestive physiology, and host‐specific microbial selection mechanisms between species [33, 34]. Nonetheless, Langon et al. [24] reported Bacteroidetes as the dominant feline phylum, indicating that phylum‐level community structure may vary among populations and study settings. Breed‐specific variation in microbial composition was evident in this study, corroborated by heatmaps of the 20 most prevalent phyla. Although the same major phyla were detected across breeds, their varying relative abundances imply a potential influence of host genetics and breed‐associated factors on the microbiome structure [32, 35]. These findings suggest that species and breed affect the relative distribution of core bacterial taxa rather than the presence of distinct microbial groups.

At the genus level, distinct differences were recorded between dogs and cats, as well as among breeds. *Bacillus*, *Lysinibacillus*, and *Brevibacillus* were enriched in dogs, with *Bacillus* particularly abundant in DLB and *Lysinibacillus* and *Brevibacillus* showing greater representation in DSG. The presence of abovementioned genera, which frequently associated with environmental sources and have been identified in canine intestinal communities, indicates a potential influence of both host and environmental factors on their distribution [36, 37]. In contrast, *Prevotella* was the dominant genus in both species but occurred more frequently in cats (66.7% vs. 49.0%), potentially reflecting dietary influences, as *Prevotella* is a key degrader of polysaccharides [38]. Breed‐specific variation was further supported by heatmap analysis of the Top 35 genera. At the species level, a large proportion of unclassified taxa was observed (91.41% in dogs, 86.02% in cats) which may be due to the limitation of 16S rRNA gene amplicon sequencing Among identified species, *C*. *stercoris* was most abundant, particularly in DRG, with heatmap analysis confirming breed‐associated species‐level variation.

This is the first approach to apply 16S rRNA‐based metagenomics to reveal the fecal microbiome in Bangladeshi pets and giving a preliminary insight into the zoonotic relevance. Some of the bacterial taxa with zoonotic relevance were observed, including *E*. *cecorum*, *Sch*. *canis*, *C*. *auriscanis*, *Cam*. *helveticus*, *S. dysgalactiae*, *Sutterella wadsworthensis*, *S. canis*, *E. casseliflavus*, and *Lawsonella clevelandensis*. Breed‐specific associations were evident: *E. cecorum*, *Sch. canis*, and *C. auriscanis* were more prevalent in DRG; *S. dysgalactiae* in CMB; and *Cam. helveticus* in CP.

Each of these taxa carries important zoonotic implications. *E. cecorum*, a commensal of multiple species, is an emerging poultry pathogen and occasional cause of opportunistic human infections, including osteomyelitis and peritonitis [39, 40]. *Sch. canis*, closely related to *Actinomyces*, is associated with bite‐wound infections in humans. *Cam. helveticus*, like *Cam. jejuni* and *Cam. upsaliensis*, is prevalent in young dogs and transmissible to humans, where it causes gastroenteritis and bacteremia [41, 42]. *C. auriscanis*, was originally isolated from clinical specimen of dogs with otitis or dermatitis rather than the gastrointestinal tract and has also been recovered from human bite‐wound infections [43–45]. Similarly, *Cam. gracilis* is primarily recognized as a constituent of oral microbiota, and has been frequently associated with periodontal environments [46]. Thus, the presence of these taxa in the feces may reflect transient gastrointestinal passage due to grooming activities or swallowed oral microbiota, rather than the representing persistent gut microbial community. *Sut. wadsworthensis*, whereas typically a commensal of the human gut, has also been identified in poultry, suggesting possible cross‐species transmission. Other detected pathogens, such as *S. dysgalactiae* and *S. canis*, are known zoonotic streptococci capable of causing invasive disease in immunocompromised humans [47, 48]. *E. casseliflavus*, though primarily an animal commensal, has been implicated in rare human infections such as endophthalmitis [49]. *L*. *clevelandensis*, a recently described anaerobe, has been associated with human abscesses but was also isolated from canine otitis, suggesting pets may serve as reservoirs [50]. Additional taxa, including *Cam. gracilis*, *Bac*. *massiliensis*, and *C. urealyticum*, were also identified. Although *Cam. gracilis* has zoonotic relevance [51], *Bac. massiliensis* and *C. urealyticum* are primarily opportunistic human pathogens without confirmed animal‐to‐human transmission. Overall, the presence of these taxa highlights the diversity of the pet gut microbiome and suggests the companion animals may harbor bacterial species with potential clinical relevance. However, 16S rRNA gene amplicon sequencing alone does not confirm pathogenicity, viability, or zoonotic transmission risk, and further study are needed to clarify epidemiological significance.

Together, these findings highlight species‐ and breed‐level variation in the feline and canine microbiome, while also identifying the zoonotic relevance of few taxa rather than a direct assessment of zoonotic risk. This dual insight into microbiome ecology and zoonotic relevance emphasizes the importance of ongoing surveillance in pets, particularly in densely populated urban environments.

## 5. Conclusion

This study provides the first comprehensive metagenomic characterization of the fecal microbiome of 12 pet dogs and 12 pet cats in Dhaka, Bangladesh, with preliminary insight into zoonotic relevance. Using 16S rRNA gene amplicon sequencing, 1,148 ASVs were identified, which were dominated by Firmicutes, Proteobacteria, Actinobacteria, and Bacteroidetes. Cats exhibited slightly greater microbial richness than dogs, with breed‐specific differences. Pet species and breed affect the relative abundance of core bacterial taxa rather than the presence of distinct microbial groups. *E*. *cecorum*, *Sch*. *canis*, *Cam*. *helveticus*, and *Sut*. *wadsworthensis* were detected as the bacterial taxa with zoonotic relevance rather than the direct assessment of zoonotic risk. However, this study has some limitations, such as the modest sample size and limited geographical coverage, and the absence of host‐ and management‐related data. Taxonomic identification was based solely on 16S rRNA gene amplicon sequencing, with no culture‐based validation, pathogen‐specific testing, or negative‐control sequencing. Therefore, bacterial taxa associated with zoonotic infections should be interpreted as having potential zoonotic relevance rather than representing confirmed zoonotic risk. Despite these limitations, the study provides baseline data on the fecal microbiome of pet dogs and cats in Bangladesh.

## Author Contributions

**Mir Nishat Tasnim Tania:** conceptualization, data curation, formal analysis, methodology, writing—original draft, and writing—review and editing; **Mirza Synthia Sabrin:** data curation, formal analysis, funding acquisition, and writing—review and editing; **Muhammad Abdul Mannan:** data curation, formal analysis, and writing—review and editing; **Md. Mominul Islam:** data curation, formal analysis, and writing—review and editing; **Muhammad Tofazzal Hossain:** data curation, formal analysis, and writing—review and editing; **Md Hafizur Rahman:** data curation, formal analysis, and writing—review and editing; **Md. Rashedul Islam:** methodology, and writing—review and editing; **Sajeda Sultana:** methodology and writing—review and editing; **Md. Shahdat Hossain:** formal analysis and writing—review and editing; **Mahfuzul Islam:** conceptualization, data curation, formal analysis, funding acquisition, investigation, methodology, validation, visualization, project administration, resources, software, supervision, writing—original draft, and writing—review and editing.

## Funding

This study was supported by the Ministry of Science and Technology, Government of the People′s Republic of Bangladesh (SRG‐241179).

## Conflicts of Interest

The authors declare no conflicts of interest.

## Data Availability

The data that support the findings of this study are openly available in National Center for Biotechnology Information (NCBI) at https://www.ncbi.nlm.nih.gov/sra/?term=PRJNA1337301 (Reference Number PRJNA1337301).
